# The effect of data resampling methods in radiomics

**DOI:** 10.1038/s41598-024-53491-5

**Published:** 2024-02-03

**Authors:** Aydin Demircioğlu

**Affiliations:** Institute of Diagnostic and Interventional Radiology and Neuroradiology, University Hospital Essen, Hufelandstraße 55, 45147 Essen, Germany

**Keywords:** Machine learning, Predictive markers

## Abstract

Radiomic datasets can be class-imbalanced, for instance, when the prevalence of diseases varies notably, meaning that the number of positive samples is much smaller than that of negative samples. In these cases, the majority class may dominate the model's training and thus negatively affect the model's predictive performance, leading to bias. Therefore, resampling methods are often utilized to class-balance the data. However, several resampling methods exist, and neither their relative predictive performance nor their impact on feature selection has been systematically analyzed. In this study, we aimed to measure the impact of nine resampling methods on radiomic models utilizing a set of fifteen publicly available datasets regarding their predictive performance. Furthermore, we evaluated the agreement and similarity of the set of selected features. Our results show that applying resampling methods did not improve the predictive performance on average. On specific datasets, slight improvements in predictive performance (+ 0.015 in AUC) could be seen. A considerable disagreement on the set of selected features was seen (only 28.7% of features agreed), which strongly impedes feature interpretability. However, selected features are similar when considering their correlation (82.9% of features correlated on average).

## Introduction

In recent years, medical image analysis has seen increased application, with radiomics emerging as a prominent technique^1–3^. Radiomics involves analyzing images by applying machine-learning techniques to quantitative characteristics extracted from imaging data, like morphological and textural features. It aids in diagnostics and predictions, such as identifying tumor molecular types^4,5^ and predicting disease outcomes^6,7^.

Radiomics models often predict rare diseases stemming from clinical routine^8,9^; consequently, the resulting datasets can often be class-imbalanced, meaning that the sample size of one class is much smaller than the other^10^. This imbalance can give rise to several modeling challenges since classifiers might overfit the majority class; that is, they model the majority class primarily and treat the minority class as noise. In this case, the utility of the classifier is largely diminished since the minority class is often predicted wrong^11^.

This problem is often tackled by balancing the data using resampling methods, which add or remove samples to obtain evenly sized classes. Resampling methods mainly fall into three categories: Oversampling, undersampling, and a combination of both^12,13^. Oversampling creates synthetic minority samples, whereas undersampling removes samples from the majority class. These strategies also have drawbacks. Since, in the context of radiomics, the sample sizes are often relatively small, undersampling could remove valuable information and thus severely affect overall performance. Oversampling, on the other hand, might generate incorrect samples and thus distort the data, leading to decreased performance as well. However, most studies in radiomics that employ resampling only evaluate it on a single dataset^14,15^; therefore, the measured effect could be specific to the dataset. In addition, resampling could also influence feature selection methods and the set of selected features, which would, in turn, affect the interpretation of the resulting models.

To measure these effects, in this study, we applied nine different resampling methods to fifteen radiomics datasets. We estimated their impact on the predictive performance and the set of selected features to gain insight into the overall effect of resampling methods on radiomic datasets.

## Results

### Predictive performance

**Figure 1:**
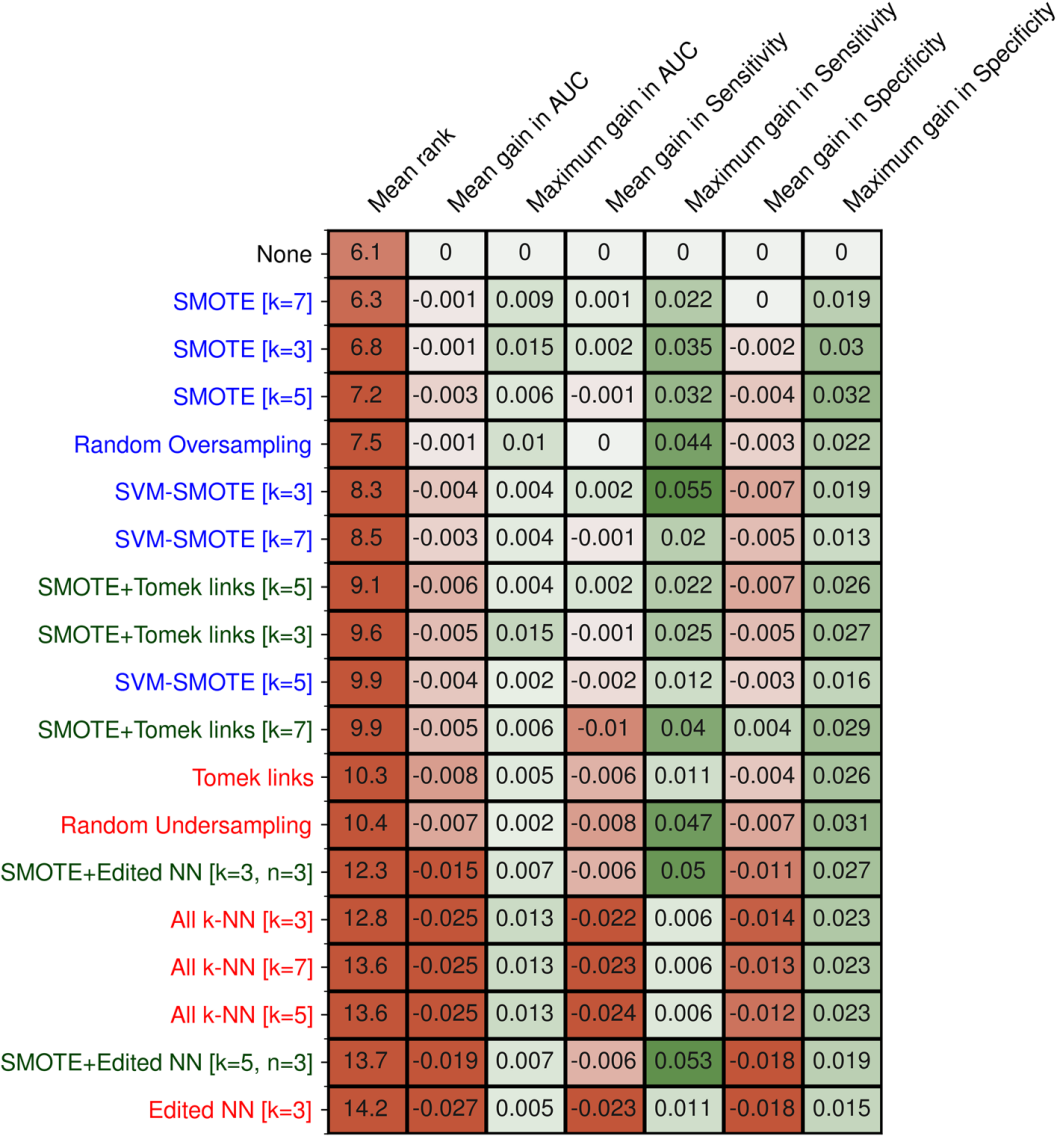
Relative predictive performance of the resampling methods. Mean rank and mean gain in AUC, sensitivity and specificity of all resampling methods across all datasets, compared to not resampling (None). Maximum gain in AUC, sensitivity, and specificity denotes the largest difference seen in any of the datasets. Oversampling methods are denoted in blue, undersampling methods in red, and combined methods in green.

Overall, no large difference in predictive performance could be seen between the resampling methods (Fig. 1), and, on average, resampling resulted in a slight loss in AUC (up to − 0.027 for the worst resampling method). Compared to not applying a resampling method, most oversampling methods (SMOTE, SVM-SMOTE) virtually showed no difference (up to + 0.015 maximum difference). Undersampling methods performed worse, especially Edited NN, and all k-NN showed losses in AUC, which showed at least a loss of at least 0.025. The same was also true for the two combined methods.

**Figure 2:**
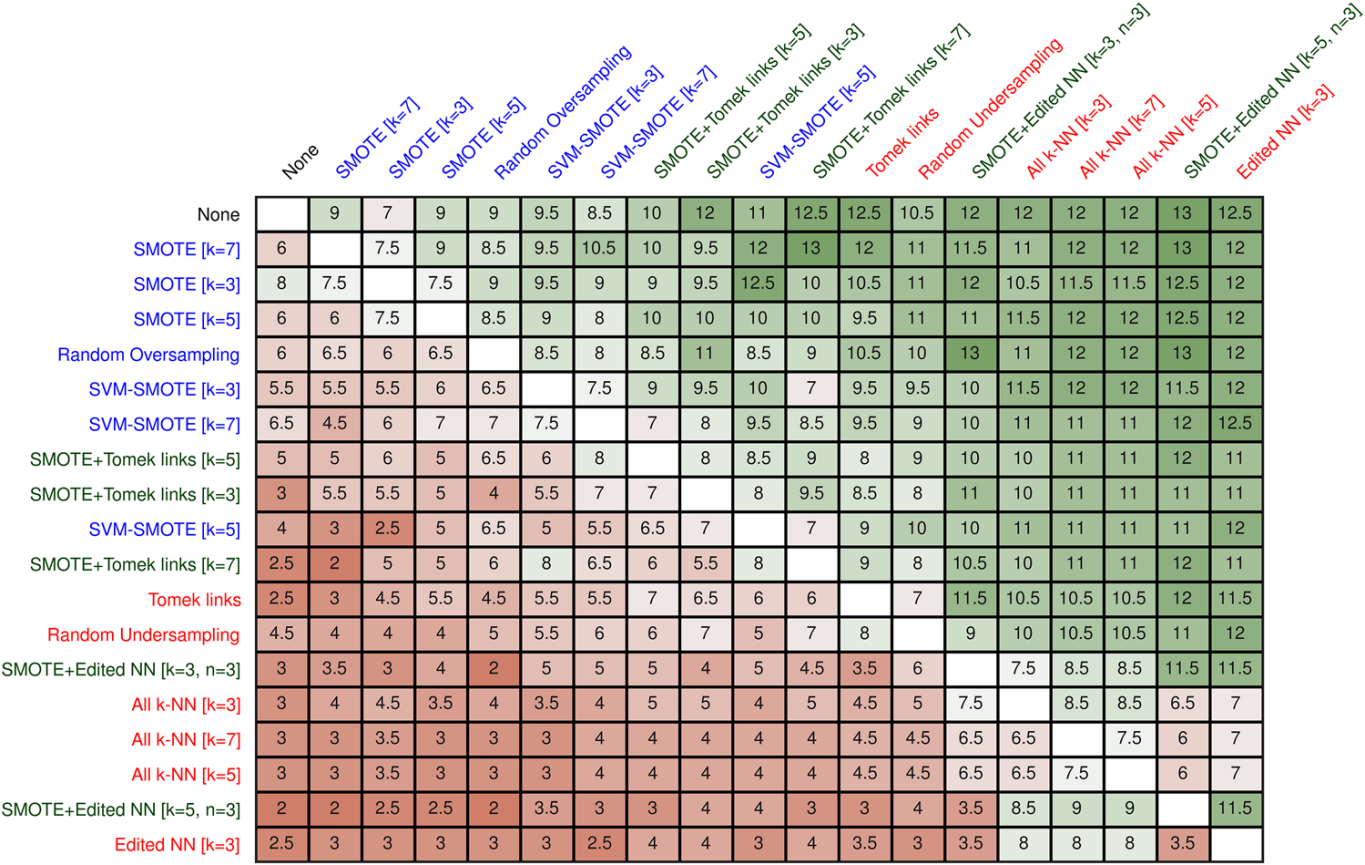
Pairwise wins and losses for all resampling methods. Wins and losses of all resampling methods. Each row denotes how often the resampling method won against the other methods (column). Draws between resampling methods counted as 0.5. Oversampling methods are denoted in blue, undersampling methods in red, and combined methods in green.

**Figure 3:**
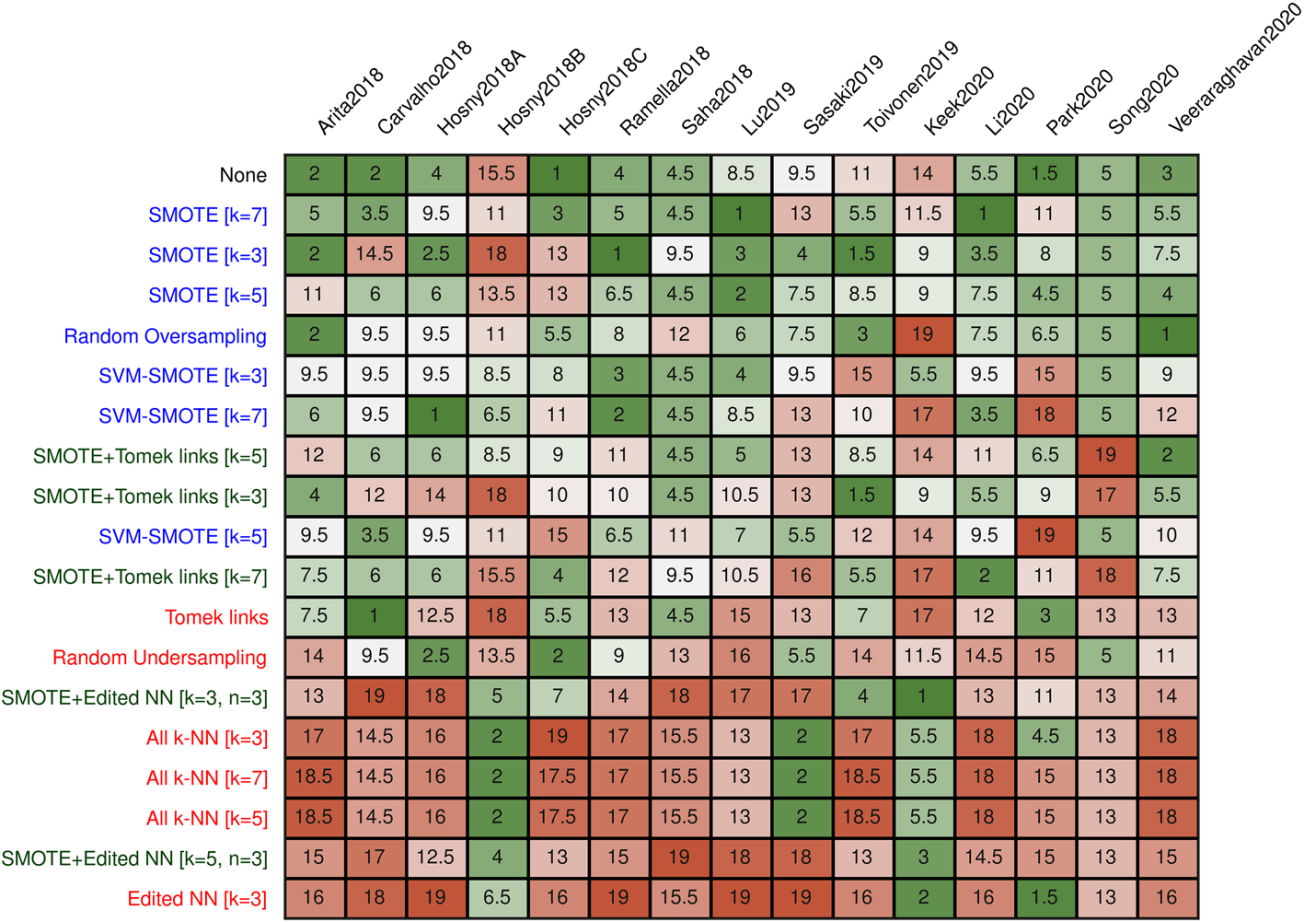
Rankings on each dataset**.** The rankings on each dataset. Rankings were obtained by sorting the AUCs of the best-performing model. Draws were counted as 0.5. Oversampling methods are denoted in blue, undersampling methods in red, and combined methods in green.

Yet, no single method outperformed all others across all datasets (Fig. 2): For example, the worst-performing method, Edited NN (k = 3) still showed a small performance increase (+ 0.013 in AUC) when compared to the best oversampling method on a dataset (Fig. 3). The Friedman test indicated a statistical significance between the resampling methods (p < 0.001); a post hoc Nemenyi test showed that the all k-NN method (with k = 5 and k = 7) were inferior to not resampling and to SMOTE (k = 7) (all p < 0.05). In addition, Edited NN (k = 3) was significantly worse than SMOTE (k = 7) (p = 0.04).

Regarding the sensitivity and specificity of the resulting models, again, no clear difference on average could be seen between the resampling methods (Fig. 1). However, in contrast to AUC, the sensitivity showed a more considerable gain on specific datasets (up to 0.055 in sensitivity). Similarly, the specificity did improve on average compared to not resampling. Nevertheless, more consistent gains of up to 0.032 could be seen for nearly all methods in specific datasets.

### Feature agreement and similarity

**Figure 4:**
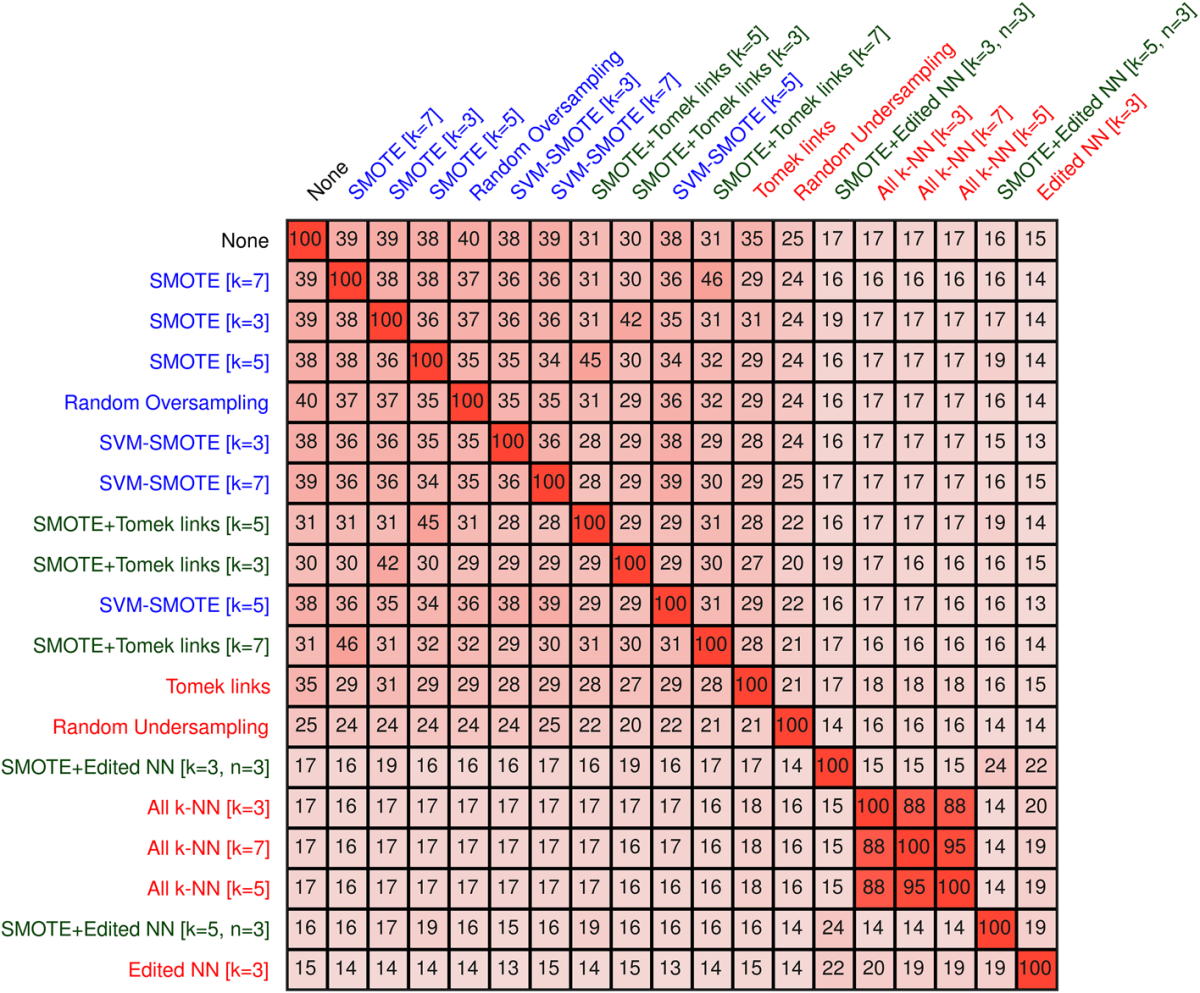
Feature agreement using Jaccard index**.** Agreement of the set of features selected by the resampling methods. For this, the Jaccard index of the selected features on each fold of the cross-validation were computed and averaged. Oversampling methods are denoted in blue, undersampling methods in red, and combined methods in green.

Resampling changed the selected features that performed best in terms of AUC (Fig. 4). Using the Jaccard index, on average, the set of selected features agreed with only 28.7%. The highest agreement between any oversampling method and no resampling at all was for random oversampling, with an agreement of 40%. Using the Ochai index did not alter these results largely. On average, a higher agreement of around 38.5% were seen (Fig. S1 in the Additional file 1). The largest feature agreement between no resampling were again observed for random oversampling.

**Figure 5:**
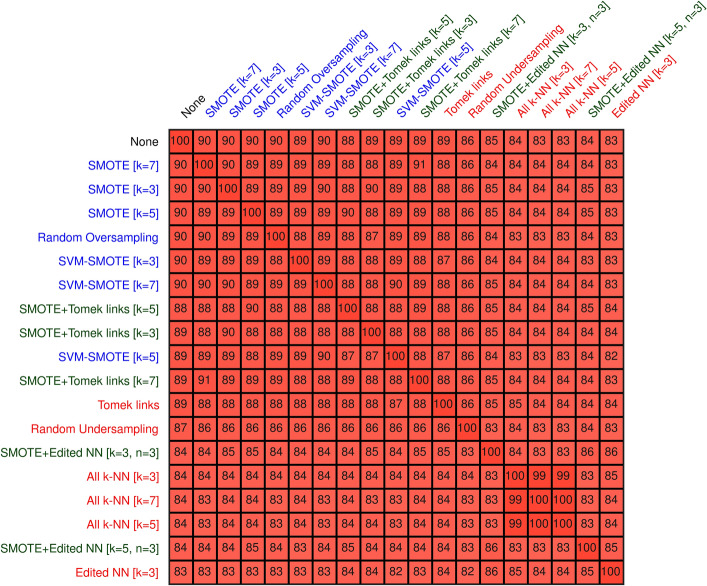
Features similarity based on correlation. Similarity among the set of features selected by the resampling methods. The similarity was computed by identifying the maximum correlated feature and averaging. Oversampling methods are denoted in blue, undersampling methods in red, and combined methods in green.

Feature similarity was higher and summed up to 86.9% (Fig. 5). Overall, it seemed the worse the resampling method performed relatively, the less similar the selected features were. Using the Zucknick measure led to even smaller feature similarities (Fig. S2 in the Additional file 1).

## Discussion

Resampling methods have often been applied in radiomics, with the promise of improving the predictive performance if the data is unbalanced. In this study, we estimated the impact of different resampling methods on the predictive performance and the selected features across multiple datasets.

Regarding the predictive performance, virtually no improvement was seen compared to not resampling. Even worse, applying undersampling decreased the performance on average. On specific datasets, however, a slight increase of up to + 0.015 in AUC could be seen for the SMOTE, showing that when only a single dataset is compared, one method can outperform every other. Yet, these observations do not generalize since SMOTE performed worse on other datasets. The same is also true for the models' sensitivity and specificity. While, on average, no improvement compared to resampling was seen, a higher sensitivity (up to + 0.055) and specificity (up to + 0.032) could be observed on specific datasets. Again, this was dependent on the dataset; it also means that both models performed worse in terms of their sensitivity and specificity on other datasets.

More complicated is the situation when comparing the agreement of the set of selected features of the best-performing models. Even if two different resampling models resulted in similarly performing models, this does not entail that the same amount or the same set of features were chosen. On average, less than one-third of the selected features agreed, which shows that if one were to identify the features with biomarkers, no agreement could be reached when models were trained using different resampling methods. Using the Ochiai index instead of the more commonly used Jaccard index to measure the agreement did not change this picture, although a higher agreement (around 10%) was observed.

However, the picture changed when similarity was considered: On average, each feature selected by the best-performing model for one resampling method correlated highly to a feature selected by another resampling method. It is partially an effect of the high correlation present in radiomic datasets^16^: Resampling can change the statistical distribution of the features so that the feature selection identifies other features as relevant, but it seems to select highly correlated features, i.e., those that contain similar information. We have employed our own measure for feature similarity since no universally accepted metric exists^17^. This measure intuitively captures the average of the highest correlations between the two feature sets. Alternatively, we have employed the Zucknick measure, which can be understood as a variant of the Jaccard index considering feature similarity. Using this measure, however, led to lower feature similarities. One reason for this difference is that the Zucknick measure takes the number of selected features into account, which our measure does not. The Zucknick measure can subsequently lead to unexpected results when features are duplicated in the feature sets. Therefore, we believe our measure is more appropriate for measuring feature similarity.

Together with the fact the predictive performance did not show improvements, this indicates that resampling in radiomic datasets does not help in radiomics as much as one would hope for.

Given the relatively large amount of radiomic studies utilizing SMOTE, or other resampling methods, our result is surprising. However, Blagus and Lusa analyzed SMOTE on three high-dimensional genetic datasets and concluded that it had no measurable effect on high-dimensional datasets and that undersampling is preferable to SMOTE^18^. Our results confirmed these observations partly. Indeed, none of the resampling methods improved the overall predictive performance, yet, SMOTE and its variants did not result in a drop in predictive performance as did undersampling methods. The difference to the study of Blagus might lie in the datasets; radiomics datasets are also high-dimensional but might have different characteristics than genetic datasets, which could lead to different behavior.

Our results could also point towards a publication bias: If resampling did not improve predictive performance, it might have been dropped from reporting, leaving only those studies where resampling did help. Arguably, this would hurt radiomic research from a scientific viewpoint^19^. Another bleak explanation could be that some studies did not apply the resampling correctly. If only cross-validation is used without an independent test set, it is of utmost importance that resampling is applied only to the training set and does not utilize the validation set in any way^20,21^. If this is not followed, a large bias can be expected^22,23^; yet, this kind of error is common^24^ and often cannot be detected without access to the code, which is most often not provided in radiomic studies. There is also the possibility that the setup of these studies differed in some ways from ours; for example, we only used filtering feature selection methods and more simple classifiers. More intricate wrapper methods like SVM-RFE combined with more complex classifiers like XgBoost might perform better in specific datasets. However, we followed rather strictly the usual radiomic pipeline and employed the most often-used methods.

Other studies partly confirm our results. Sarac and Guvenis considered six different resampling methods in a cohort of patients with oropharyngeal cancer to determine their HPV status^25^. They demonstrated that oversampling performed better overall than undersampling, with SMOTE obtaining the highest performance. While in our experiments, oversampling performed on average better than undersampling, and SMOTE performed relatively well, not resampling at all performed best. Unfortunately, Sarac and Guvenis did consider this. In a similar study, Zhang et al. tested four subsampling methods in a cohort of patients with non-small-cell lung cancer (NSCLC) and reported that applying SMOTE improved the performance significantly, with an improvement of + 0.03 in AUC^26^, while other resampling methods seemed to fare less well. However, it must be noted that they extracted only 30 features, which makes their data low-dimensional (more samples than features). Therefore, the improvement might be larger than we observed on a single dataset. In a recent large benchmarking study on non-radiomics datasets, Tarawneh et al. concluded that many resampling methods are not helpful^27^. This result is in line with our study, even though they employed only low-dimensional datasets with very high imbalance, which is often uncommon in radiomics.

In our study, we applied resampling before feature selection, following the observations from Blagus and Lusa^18^. Yet, resampling can be used before and after feature selection, and there are arguments for both choices. Since feature selection methods might be affected strongly by imbalance, applying resampling ahead might be more beneficial^18^. However, using it after feature selection also has some advantages: As the data set is slimmed down by feature selection, the resampling approach will be computationally more effective and will not resample otherwise irrelevant features. Yet, the situation is not clear: In a recent study on high-dimensional genetic data, Ramos-Pérez et al.^28^ demonstrated that the order of resampling and feature selection could depend on the resampling method. They state that random undersampling (RUS) should be ideally performed before feature selection, but random oversampling (ROS) and SMOTE afterward. This result was not observed in our study, where SMOTE applied upfront outperformed RUS. Accordingly, when confronted with a new dataset, both variants should be tested if predictive performance is the goal.

Some limitations apply to our study. First, although we have employed a rather large collection of datasets, these were collected opportunistically. We cannot exclude that a potential bias might be present. Furthermore, we could not use external data since there are only very few publicly available datasets where such data is provided. We cannot rule out that resampling methods could help the model to generalize better to external data^29^. Instead, we utilized cross-validation, which can measure robustness with respect to different distributions only in a limited way. In addition, cross-validation could lead to some overfitting. However, this would possibly affect all methods by a similar amount. Due to restricted computational resources, we opted for a fivefold cross-validation with 30 repeats, although we acknowledge that other validation schemes like leave-one-out CV or using a higher number of repeats could allow for more precise results. In addition, although we tested the most commonly used resampling methods, many more have been developed, especially methods based on generative adversarial networks, which are promising^30^.

The same applies to the feature selection methods and classifiers we employed in this study. We also only considered generic features, that is, those based on morphological, intensity, and textural features, and did not employ datasets with features extracted from deep neural networks^31–33^. Since these features might be quantitatively different, our conclusions might not hold for these datasets. Furthermore, we did not apply feature reduction, like principal component analysis, because these methods generate new features which usually do not have a direct interpretation. However, since features are thought to correlate to biomarkers, their interpretation is critical in radiomics. Also, our study only considered AUC as the primary metric for predictive performance and considered sensitivity and specificity as secondary metrics. Depending on the problem, other metrics can be more important. Our study cannot estimate the effect of resampling on these metrics. However, AUC is arguably the most essential metric since it can be considered as the de-facto metric for radiomic studies^34,35^.

Our study demonstrated that, on average, resampling methods did not improve the overall predictive performance of models in radiomics, although this might be the case for a specific dataset. Applying resampling largely changed the set of selected features, which obstructs feature interpretation. However, the set of features was highly correlated, indicating that resampling does not change the information in the data by much.

## Methods

In this study, we utilized previously published and publicly accessible datasets to ensure reproducibility. The corresponding ethical review boards granted ethical approval for these datasets. Since the study was retrospective, the local Ethics Committee (Ethik-Kommission, Medizinische Fakultät der Universität Duisburg-Essen, Germany) waived the need for additional ethical approval. The study was conducted following relevant guidelines and regulations.

### Datasets

We collected a total of 15 publicly available radiomic datasets (Table 1). These datasets were not collected systematially but were gathered opportunistically, reflecting the scarcity of relevant data in the field^36^. All datasets comprised already extracted radiomics features; no feature generation was performed for this study. Each dataset was prepared by removing non-radiomic features (like clinical or genetic data) before merging all data splits. The datasets were all high-dimensional, meaning they had more features than samples, except for two datasets (Carvalho2018 and Saha2018).

**Table 1:** Overview of the datasets used.

Dataset	N	d	N+	N−	Balance	Modality	Tumor type	DOI
Arita2018^49^	168	685	111	57	1.9	MRI	Brain	10.1038/s41598-018-30273-4
Carvalho2018^50^	262	118	154	108	1.4	FDG + PET	NSCLC	10.1371/journal.pone.0192859
Hosny2018A ^51^	293	985	159	134	1.2	CT	NSCLC	10.1371/journal.pmed.1002711
Hosny2018B^51^	211	1005	60	151	2.5	CT	NSCLC	10.1371/journal.pmed.1002711
Hosny2018C^51^	183	1005	133	50	2.7	CT	NSCLC	10.1371/journal.pmed.1002711
Ramella2018^52^	91	243	50	41	1.2	CT	NSCLC	10.1371/journal.pone.0207455
Saha2018^53^	922	530	327	595	1.8	DCE-MRI	Breast	10.1038/s41416-018-0185-8
Lu2019^54^	213	658	91	122	1.3	CT	Ovarian cancer	10.1038/s41467-019-08718-9
Sasaki2019^55^	138	588	68	70	1.0	MRI	Brain	10.1038/s41598-019-50849-y
Toivonen2019^56^	100	7106	80	20	4.0	MRI	Prostate cancer	10.1371/journal.pone.0217702
Keek2020^57^	273	1323	119	154	1.3	CT	HNSCC	10.1371/journal.pone.0232639
Li2020^58^	51	397	32	19	1.7	MRI	Glioma	10.1371/journal.pone.0227703
Park2020^59^	768	941	183	585	3.2	US	Thyroid cancer	10.1371/journal.pone.0227315
Song2020^60^	260	265	127	133	1.0	MRI	Prostate cancer	10.1371/journal.pone.0237587
Veeraraghavan2020^61^	150	201	47	103	2.2	DCE-MRI	Breast	10.1038/s41598-020-72475-9

Only publicly available datasets were used. N denotes the sample size, d the number of features, N+ the number of positive samples, N− the number of negative samples, B is the ratio of the majority class to the minority class. DOI is the digital object identifier of the publication corresponding to the dataset.

### Preprocessing

A few missing values were observed in the datasets, notably in the three datasets by Hosny et al. Here, at most, 0.79%, 0.65% and 0.19% of the values were missing. However, these missings nearly exclusively affected the two features, ‘exponential_ngtdm_contrast’ and ‘exponential_glcm_correlation’; the missings possibly occurred because of numerical overflows due to the exponential function. These two features were consequently removed from the analysis. Other datasets had less than < 0.16% missing values. Due to how radiomics is computed, these values were likely missing completely at random and did not lead to systematic bias. The missing values were removed by imputing them with feature means. All datasets were then normalized by z-Score, i.e., by subtracting the mean of each feature and dividing by the standard deviation.

### Resampling methods

Nine different resampling methods were used in this study, encompassing over- and undersampling and combination techniques (Table 2). The undersampling methods, which were random undersampling, edited nearest neighborhood (ENN), all k-NN, and Tomek links, aim to reduce the size of the majority class to match that of the minority class. In contrast, the oversampling methods, random oversampling, synthetic minority oversampling technique (SMOTE), and SVM-SMOTE, aim to increase the size of the minority class to match that of the majority class. Combination methods, like SMOTE + ENN, and SMOTE + Tomek, involve resampling both classes, usually resulting in datasets where the majority class is smaller than before, and the minority class becomes larger.

**Table 2:** List of resampling methods and parameters.

Method	Parameters	Type
Random undersampling	–	Undersampling
Edited NN	k = 3	Undersampling
All k-NN	k = 3, 5, 7	Undersampling
Tomek links	–	Undersampling
Random oversampling	–	Oversampling
SMOTE	k = 3, 5, 7	Oversampling
SVM-SMOTE	k = 3, 5, 7	Oversampling
SMOTE + edited NN	k = 3, 5, n = 3	Combined
SMOTE + Tomek links	k = 3, 5, 7	Combined
None	–	–

Some resampling methods need a choice of the neighborhood size to consider during the resampling, In the original study SMOTE^37^, the neighborhood size was set to 5. Since this choice might not be optimal for all 15 datasets, in addition, a smaller and a larger size was also considered, i.e., the neighborhood size was chosen from 3, 5, and 7. However, in a few datasets, a size of 5 or 7 for undersampling methods effectively removed the minority class; therefore, in ENN and SMOTE + ENN, only a neighborhood of size 3 was used.

### Feature selection

For the selection of relevant features, four often-used feature selection methods were used^38^: Analysis of variance (ANOVA), Bhattacharyya scores, Extra trees (ET), and the least absolute shrinkage and selection operator (LASSO). Being filter methods, each of them scored the features according to their estimated relevance. The highest-scoring features were then extracted based on a choice of how many features should be included. Here, the number of selected features was chosen on a logarithmic scale among N = 1, 2, 4, … 32, 64. This approach allowed for efficient exploration while maintaining low computational complexity.

### Classifiers

Models were trained using often-used classifiers^39^: k-Nearest Neighbor (kNN), logistic regression (LR), naive Bayes, random forest (RF), and kernelized SVM (RBF-SVM). These methods had partly hyperparameters, e.g., in the case of the RBF-SVM, it is known that its performance depends strongly on the choice of the regularization parameter C^40^. This parameter was therefore optimized using a simple grid search on the training data^41,42^, during which it was selected from 2^–10^, 2^–8^, …, 2^–1^, 1, 2^2^, … 2^8^, 2^10^. The kernel width γ of the RBF-SVM was set to the inverse of the mean distance between any two samples. For the RF, the number of trees was set to 250. The neighborhood size of the k-NN was chosen among 1, 3, 5, 7, 9. Finally, the regularization parameter of the logistic regression was also chosen from 2^–10^, 2^–8^, …, 2^–1^, 1, 2^2^, … 2^8^, 2^10^. Other parameters were left at their default values.

### Training

**Figure 6:**
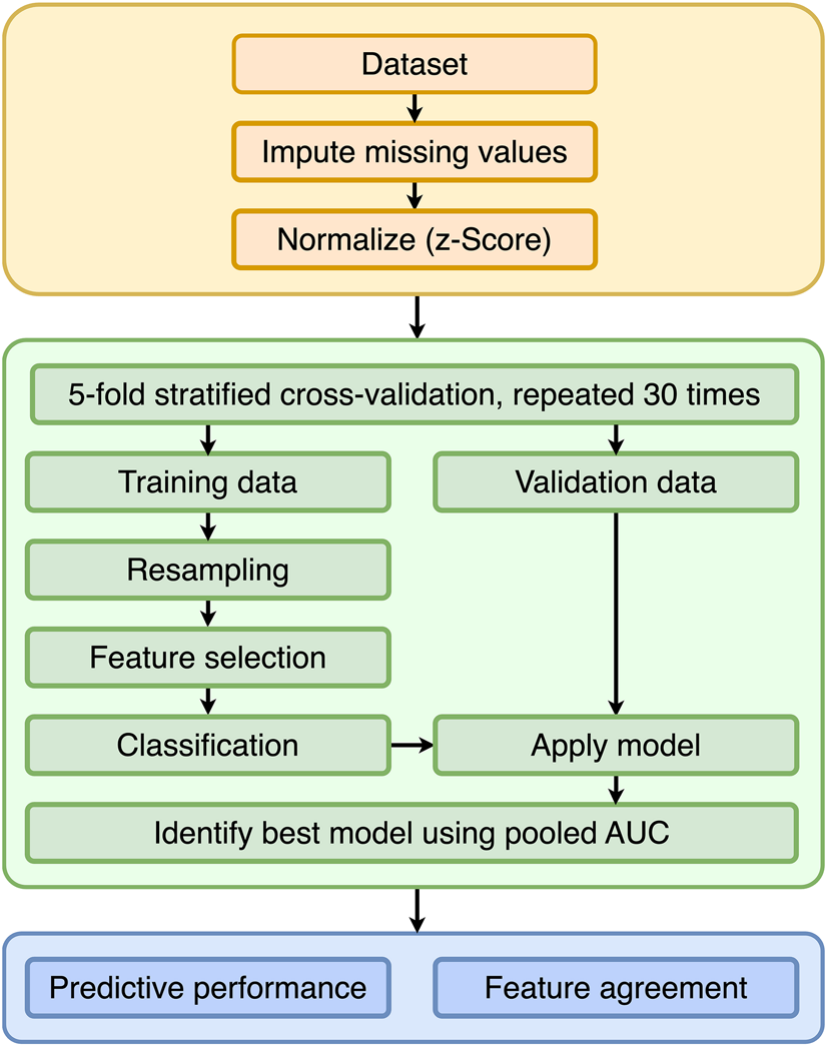
Flow chart of the experiments.

The evaluation followed the standard radiomics pipeline^43,44^ and was performed using a fivefold stratified cross-validation (CV) with 30 repeats (Fig. 6). Stratification was employed to ensure that the original class balance of the data is kept in the test folds as well. In each repeat, first, the data was split into five folds. In turn, each fold was once left out for validation, while the other four folds were used as a training set. It was then resampled using one of the resampling methods. A feature selection method and a classifier were subsequently applied to the resulting data. The final model was then evaluated on the validation fold, i.e., the relevant features were selected in the validation fold first, and then prediction took place with the classifier.

### Predictive performance

Since the primary focus in radiomics is obtaining accurate predictions, the macro-averaged area under the receiver operator characteristic curve (AUC) over the five CV validation folds was used to identify the best-performing model. The best-performing models were then analyzed; models performing worse were discarded. In addition, the sensitivity and the specificity of the models were computed as secondary metrics.

### Feature agreement and similarity

The agreement of the selected features was compared pairwise for all resampling methods over each training fold during the CV. We used the Jaccard index, also called Intersection-over-Union, to measure agreement. Since no universal metric exists, we also employed the Ochiai index^45^.

Since radiomic datasets are known to be highly correlated^16^, two sets of features might look vastly different, although they might describe similar information. Therefore, we computed the similarity between the set of selected features. It is calculated roughly as follows: First, for each feature in the one set, the feature with the highest correlation in the other set is identified. The (symmetrized) mean over all these correlations is then defined as the similarity. More information can be found in Additional file 1. Since this is an ad-hoc metric, we also computed the Zucknick measure^46^, which can be understood as a correlation-corrected version of the Jaccard index^45^.

### Software

Experiments were implemented using Python 3.10. The resampling methods were utilized from the Imbalanced-learn package 0.10.0^47^. Our code repository can be found on github, where, apart from the results, a complete list of the dependencies and software versions used in this study can also be found.

### Statistics

Descriptive statistics were reported using mean and standard deviation. P-values below 0.05 were considered statistically significant. All statistics were computed using Python 3.10. Resampling methods were compared using a Friedman test and a post hoc Nemenyi test^48^. The Friedman test was preferred over the ANOVA test since it is a non-parametric test and has thus fewer assumptions on the data. Since the Friedman test only tests for the hypothesis of whether there are any differences between the methods, a pairwise post hoc Nemeyi test was employed to determine the differences. The Nemenyi test can be understood as the non-parametric equivalent of the Tukey test usually employed for the ANOVA test^48^.

### Ethics approval and consent to participate

This is a retrospective study using only previously published and publicly accessible datasets. The ethical approval for this study was waived by the local Ethics Committee (Ethik-Kommission, Medizinische Fakultät der Universität Duisburg-Essen, Germany) due to its retrospective nature.

### Supplementary Information

Supplementary Information.

## Data Availability

All datasets are publicly available. Code, data and results can be found on the public repository at https://www.github.com/aydindemircioglu/radResampling.

## References

[1] Aerts HJWL (2014). Decoding tumour phenotype by noninvasive imaging using a quantitative radiomics approach. Nat. Commun..

[2] Afshar P, Mohammadi A, Plataniotis KN, Oikonomou A, Benali H (2019). From handcrafted to deep-learning-based cancer radiomics: Challenges and opportunities. IEEE Signal Process. Mag..

[3] Mayerhoefer ME (2020). Introduction to radiomics. J. Nucl. Med..

[4] Li W, Yu K, Feng C, Zhao D (2019). Molecular subtypes recognition of breast cancer in dynamic contrast-enhanced breast magnetic resonance imaging phenotypes from radiomics data. Comput. Math. Methods Med..

[5] Cho N (2020). Imaging features of breast cancer molecular subtypes: State of the art. J. Pathol. Transl. Med..

[6] Fave X (2017). Delta-radiomics features for the prediction of patient outcomes in non-small cell lung cancer. Sci. Rep..

[7] Lucia F (2018). Prediction of outcome using pretreatment 18F-FDG PET/CT and MRI radiomics in locally advanced cervical cancer treated with chemoradiotherapy. Eur. J. Nucl. Med. Mol. Imaging.

[8] Peeken JC (2019). CT-based radiomic features predict tumor grading and have prognostic value in patients with soft tissue sarcomas treated with neoadjuvant radiation therapy. Radiother. Oncol..

[9] Suarez-Ibarrola R, Basulto-Martinez M, Heinze A, Gratzke C, Miernik A (2020). Radiomics applications in renal tumor assessment: A comprehensive review of the literature. Cancers.

[10] Tasci E, Zhuge Y, Camphausen K, Krauze AV (2022). Bias and class imbalance in oncologic data: Towards inclusive and transferrable AI in large scale oncology data sets. Cancers.

[11] Cortes, C. & Mohri, M. AUC optimization vs. error rate minimization. in *Advances in Neural Information Processing Systems*, vol. 16 (MIT Press, 2003).

[12] Batista GE, Prati RC, Monard MC (2004). A study of the behavior of several methods for balancing machine learning training data. ACM SIGKDD Explor. Newsl..

[13] Batista GE, Bazzan AL, Monard MC (2003). Balancing training data for automated annotation of keywords: A case study. Wob.

[14] Kawaji K (2023). Application of machine learning analyses using clinical and [18F]-FDG-PET/CT radiomic characteristics to predict recurrence in patients with breast cancer. Mol. Imaging Biol..

[15] Kawahara D (2021). Prediction of radiation pneumonitis after definitive radiotherapy for locally advanced non-small cell lung cancer using multi-region radiomics analysis. Sci. Rep..

[16] Demircioğlu A (2022). Evaluation of the dependence of radiomic features on the machine learning model. Insights Imaging.

[17] Bommert A, Rahnenführer J, Nicosia G (2020). Adjusted measures for feature selection stability for data sets with similar features. Machine Learning, Optimization, and Data Science.

[18] Blagus R, Lusa L (2013). SMOTE for high-dimensional class-imbalanced data. BMC Bioinform..

[19] Buvat I, Orlhac F (2019). The dark side of radiomics: On the paramount importance of publishing negative results. J. Nucl. Med..

[20] Wang L (2023). MRI-based pre-radiomics and delta-radiomics models accurately predict the post-treatment response of rectal adenocarcinoma to neoadjuvant chemoradiotherapy. Front. Oncol..

[21] Dunn B, Pierobon M, Wei Q (2023). Automated classification of lung cancer subtypes using deep learning and CT-scan based radiomic analysis. Bioengineering.

[22] Demircioğlu A (2021). Measuring the bias of incorrect application of feature selection when using cross-validation in radiomics. Insights Imaging.

[23] Samala RK, Chan H-P, Hadjiiski L, Helvie MA (2021). Risks of feature leakage and sample size dependencies in deep feature extraction for breast mass classification. Med. Phys..

[24] Desaire H (2022). How (not) to generate a highly predictive biomarker panel using machine learning. J. Proteome Res..

[25] Sarac K, Guvenis A, Rojas I, Valenzuela O, Rojas Ruiz F, Herrera LJ, Ortuño F (2023). Determining HPV status in patients with oropharyngeal cancer from 3D CT images using radiomics: Effect of sampling methods. Bioinformatics and Biomedical Engineering.

[26] Zhang Y, Oikonomou A, Wong A, Haider MA, Khalvati F (2017). Radiomics-based prognosis analysis for non-small cell lung cancer. Sci. Rep..

[27] Tarawneh AS, Hassanat AB, Altarawneh GA, Almuhaimeed A (2022). Stop oversampling for class imbalance learning: A review. IEEE Access.

[28] Ramos-Pérez I, Arnaiz-González Á, Rodríguez JJ, García-Osorio C (2022). When is resampling beneficial for feature selection with imbalanced wide data?. Expert Syst. Appl..

[29] Wang T (2023). A CT-based radiomics nomogram for distinguishing between malignant and benign Bosniak IIF masses: A two-centre study. Clin. Radiol..

[30] Hameed MAB, Alamgir Z (2022). Improving mortality prediction in acute pancreatitis by machine learning and data augmentation. Comput. Biol. Med..

[31] Li Y (2022). Molecular subtyping of diffuse gliomas using magnetic resonance imaging: Comparison and correlation between radiomics and deep learning. Eur. Radiol..

[32] Braghetto A, Marturano F, Paiusco M, Baiesi M, Bettinelli A (2022). Radiomics and deep learning methods for the prediction of 2-year overall survival in LUNG1 dataset. Sci. Rep..

[33] Demircioğlu A (2022). Predictive performance of radiomic models based on features extracted from pretrained deep networks. Insights Imaging.

[34] Le VH (2023). Development and validation of CT-based radiomics signature for overall survival prediction in multi-organ cancer. J. Digit. Imaging.

[35] Nguyen, H. S. *et al.* Predicting EGFR mutation status in non-small cell lung cancer using artificial intelligence: A systematic review and meta-analysis. *Acad. Radiol.* (2023). 10.1016/j.acra.2023.03.040 37120403

[36] Akinci D’Antonoli T, Cuocolo R, Baessler B, Pinto dos Santos D (2023). Towards reproducible radiomics research: Introduction of a database for radiomics studies. Eur. Radiol..

[37] Chawla, N. V., Lazarevic, A., Hall, L. O. & Bowyer, K. W. SMOTEBoost: Improving prediction of the minority class in boosting. in *Knowledge Discovery in Databases: PKDD 2003* (eds. Lavrač, N., Gamberger, D., Todorovski, L. & Blockeel, H.) vol. 2838, 107–119 (Springer, 2003).

[38] Demircioğlu A (2022). Benchmarking feature selection methods in radiomics. Invest. Radiol..

[39] Song J (2020). A review of original articles published in the emerging field of radiomics. Eur. J. Radiol..

[40] Chang C-C, Lin C-J (2011). LIBSVM: A library for support vector machines. ACM Trans. Intell. Syst. Technol. TIST.

[41] Bischl B (2023). Hyperparameter optimization: Foundations, algorithms, best practices, and open challenges. WIREs Data Min. Knowl. Discov..

[42] Alpaydin E (2020). Introduction to Machine Learning.

[43] Koçak B, Durmaz EŞ, Ateş E, Kılıçkesmez Ö (2019). Radiomics with artificial intelligence: A practical guide for beginners. Diagn. Interv. Radiol..

[44] Lambin P (2012). Radiomics: Extracting more information from medical images using advanced feature analysis. Eur. J. Cancer.

[45] Bommert A, Rahnenführer J, Lang M (2017). A multicriteria approach to find predictive and sparse models with stable feature selection for high-dimensional data. Comput. Math. Methods Med..

[46] Zucknick M, Richardson S, Stronach EA (2008). Comparing the characteristics of gene expression profiles derived by univariate and multivariate classification methods. Stat. Appl. Genet. Mol. Biol..

[47] Lemaître G, Nogueira F, Aridas CK (2017). Imbalanced-learn: A python toolbox to tackle the curse of imbalanced datasets in machine learning. J. Mach. Learn. Res..

[48] Demšar J (2006). Statistical comparisons of classifiers over multiple data sets. J. Mach. Learn. Res..

[49] Arita H (2018). Lesion location implemented magnetic resonance imaging radiomics for predicting IDH and TERT promoter mutations in grade II/III gliomas. Sci. Rep..

[50] Carvalho S (2018). 18F-fluorodeoxyglucose positron-emission tomography (FDG-PET)-Radiomics of metastatic lymph nodes and primary tumor in non-small cell lung cancer (NSCLC): A prospective externally validated study. PLoS ONE.

[51] Hosny A (2018). Deep learning for lung cancer prognostication: A retrospective multi-cohort radiomics study. PLOS Med..

[52] Ramella S (2018). A radiomic approach for adaptive radiotherapy in non-small cell lung cancer patients. PLoS ONE.

[53] Saha A (2018). A machine learning approach to radiogenomics of breast cancer: A study of 922 subjects and 529 DCE-MRI features. Br. J. Cancer.

[54] Lu H (2019). A mathematical-descriptor of tumor-mesoscopic-structure from computed-tomography images annotates prognostic- and molecular-phenotypes of epithelial ovarian cancer. Nat. Commun..

[55] Sasaki T (2019). Radiomics and MGMT promoter methylation for prognostication of newly diagnosed glioblastoma. Sci. Rep..

[56] Toivonen J (2019). Radiomics and machine learning of multisequence multiparametric prostate MRI: Towards improved non-invasive prostate cancer characterization. PLOS ONE.

[57] Keek S (2020). Computed tomography-derived radiomic signature of head and neck squamous cell carcinoma (peri)tumoral tissue for the prediction of locoregional recurrence and distant metastasis after concurrent chemo-radiotherapy. PLoS ONE.

[58] Li J (2020). High-order radiomics features based on T2 FLAIR MRI predict multiple glioma immunohistochemical features: A more precise and personalized gliomas management. PLoS ONE.

[59] Park VY (2020). Radiomics signature for prediction of lateral lymph node metastasis in conventional papillary thyroid carcinoma. PLoS ONE.

[60] Song Y (2020). FeAture explorer (FAE): A tool for developing and comparing radiomics models. PLoS ONE.

[61] Veeraraghavan H (2020). Machine learning-based prediction of microsatellite instability and high tumor mutation burden from contrast-enhanced computed tomography in endometrial cancers. Sci. Rep..

