# Supplementary material for: The effect of data resampling methods in radiomics
Source: Sci Rep. 2024 Feb 3;14:2858. doi: 10.1038/s41598-024-53491-5 (PMC10838284; doi:10.1038/s41598-024-53491-5)
Supplement: Supplementary file 1 — Supplementary Information. [file 41598_2024_53491_MOESM1_ESM.docx]

**The effect of data resampling methods in radiomics**

**Supplementary Information 1**

***Information on the datasets***

The datasets were collected opportunistically. All preprocessing code can be found in the github repository.

*Arita2018*

Patient collective: Patients with grade II/III glioma

Imaging: T1w, T1w+contrast, T2w, and FLAIR

Outcome: Status of IDH1/2

*Carvalho2018*

Patient collective: Patients with NSCLC in stage I-IIIB

Imaging: FDG-PET+CT

Outcome used: Mortality after 2 years

*Hosny2018A (HarvardRT)*

Patient collective: Patients with NSCLC in stage I–IIIb

Imaging: CT with and without contrast

Outcome used: Mortality after 2 years

*Hosny2018B (Maastro)*

Patient collective: Patients with NSCLC in stage I–IIIb

Imaging: CT with and without contrast

Outcome used: Mortality after 2 years

*Hosny2018C (Moffitt)*

Patient collective: Patients with NSCLC stage I–IIIb

Imaging: CT with and without contrast

Outcome used: Mortality after 2 years

*Ramella2018*

Patient collective: Patients with NSCLC in stage III treated with concurrent chemoradiation

Imaging: CT without contrast

Outcome used: Adaptive radiotherapy

*Saha2018*

Patient collective: Patients with invasive breast cancer

Imaging: Non-fat saturated T1w, fat-saturated gradient echo T1w, post-contrast T1w

Outcome used: Molecular subtype

*Lu2019*

Patient collective: Patients with epithelial ovarian cancer

Imaging: CT with contrast

Outcome used: Progression free survival after 2 years

*Sasaki2019*

Patient collective: Patients with glioblastoma

Imaging: Gd-T1w, T1w, T2w

Outcome used: pMGMT methylation status

*Toivonen2019*

Patient collective: Patients with prostate scheduled for laparoscopic prostatectomy

Imaging: GraSE T2w, TSE T2w, DWI

Outcome used: Gleason score risk group

*Keek2020*

Patient collective: Patients with stage III and IV HPV-negative HNSCC

Imaging: CT with contrast

Outcome used: Survival after 3 years

*Li2020*

Patient collective: Patients with glioma

Imaging: T2 FLAIR

Outcome used: Based on Ki-67, S-100, vimentin and CD34 immunohistochemical results (called Label in the study)

Park2020

Patient collective: Patients with histologically confirmed conventional papillary thyroid carcinoma

Imaging: Ultrasound

Outcome used: Lateral lymph node metastasis

*Song2020*

Patient collective: Patients with prostate cancer

Imaging: TSE T2w, DWI, ADC

Outcome used: Clinical significance of prostate cancer

*Veeraraghavan2020*

Patient collective: Patients with breast cancer

Imaging: CT with contrast

Outcome used: High tumor mutational burden (> 15.5 mutations per megabase)

### Resampling in the original studies

In the 15 studies which provided the data for this study, only two used resampling methods: In Li2020, SMOTE was employed, while in Veeraraghavan2020 an unspecified upsampling method was utilized.

### Similarity

The similarity between two set of features does not only consider if the same features are present in both sets but also if the selected features are correlated. For example, volume could be highly correlated to diameter, and one model might select volume, while the other select diameter. In this case, different features would be selected, but since they contain correlated information, these two sets can be regarded as similar.

Similarity is computed here as follow. Given two binary vectors of selected features $f^{A}$ and $f^{B}$ corresponding to the features of model A and model B, for each selected feature u in $f^{A}$, we compute all its correlations to selected features v in $f^{B}$. The correlation of u is then defined by the maximum Pearson correlation: $uCorr \left( u, f^{B} \right)=\max_{v \in f^{B}} Pearson (u, v)$. The correlation of $f^{A}$ and $f^{B}$ is then given by averaging all correlations of selected features A.

$$uCorr \left( f^{A}, f^{B} \right)= \frac{1}{|f^{A}|}\sum_{u \in f^{A}} uCorr (u, f^{B})$$

In other words, first, for each feature of A the maximally correlated feature in B is identified and their Pearson correlation is computed. Then, the correlation of A and B is given by the average correlation over all selected features. For example, a value of 0.5 would mean that either each feature of A can be correlated to a feature of B with a correlation of 0.5, or it could mean for half of the features a “perfect match”, i.e., a feature with correlation 1.0, can be found. Note that by definition the correlation is not symmetric. To symmetrize, we simply take the mean over both correlations, i.e.

$$Corr \left( f^{A}, f^{B} \right)= \frac{1}{2}\left( uCorr\left( f^{A}, f^{B} \right)+uCorr \left( f^{B}, f^{A} \right) \right)$$

### Feature agreement using the Ochiai index


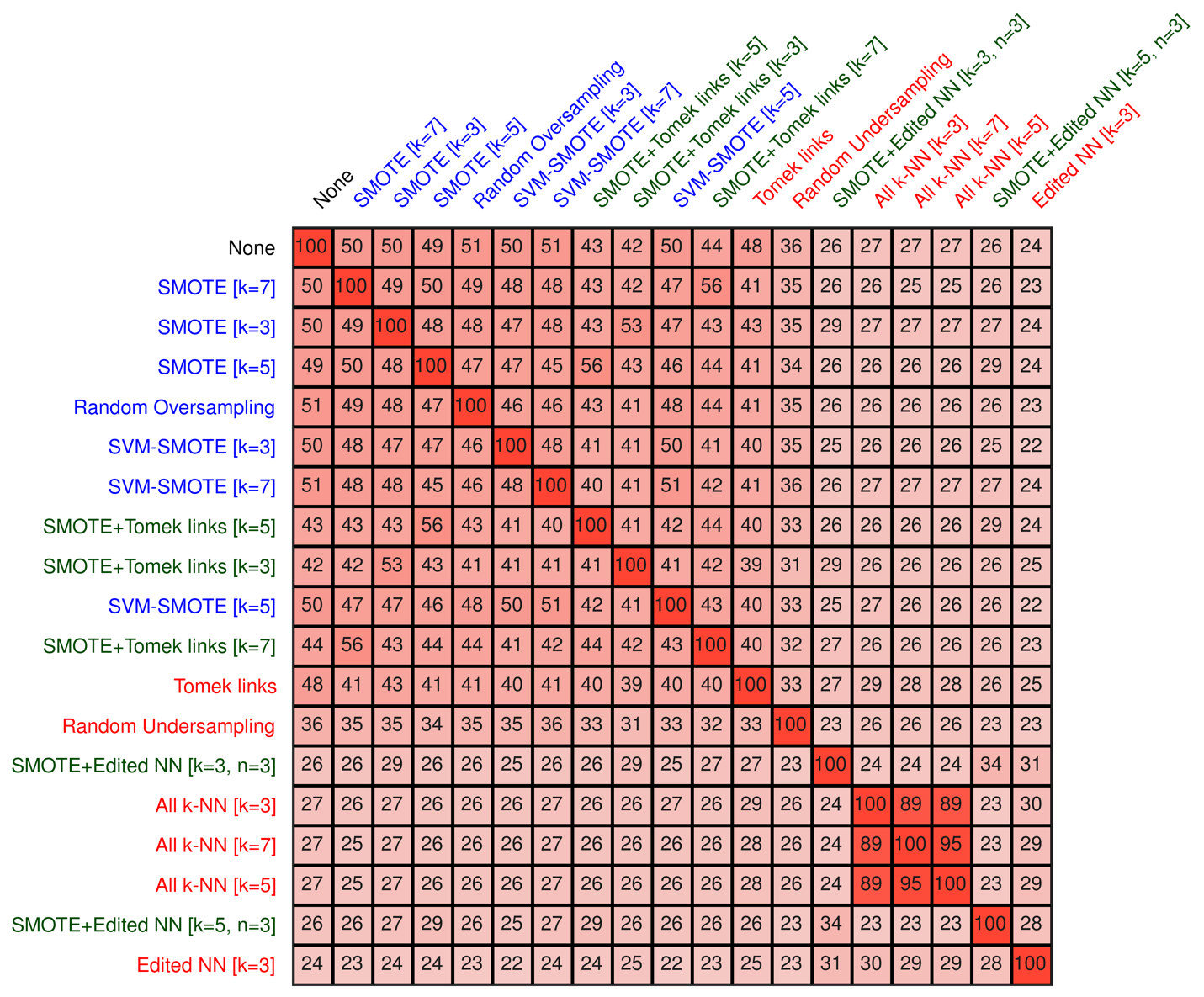


**Figure S1** Feature agreement using Ochiai index. Agreement of the set of features selected by the resampling methods. For this, the Ochiai index of the selected features on each fold of the cross-validation were computed and averaged

### Feature similarity using the Zucknick index


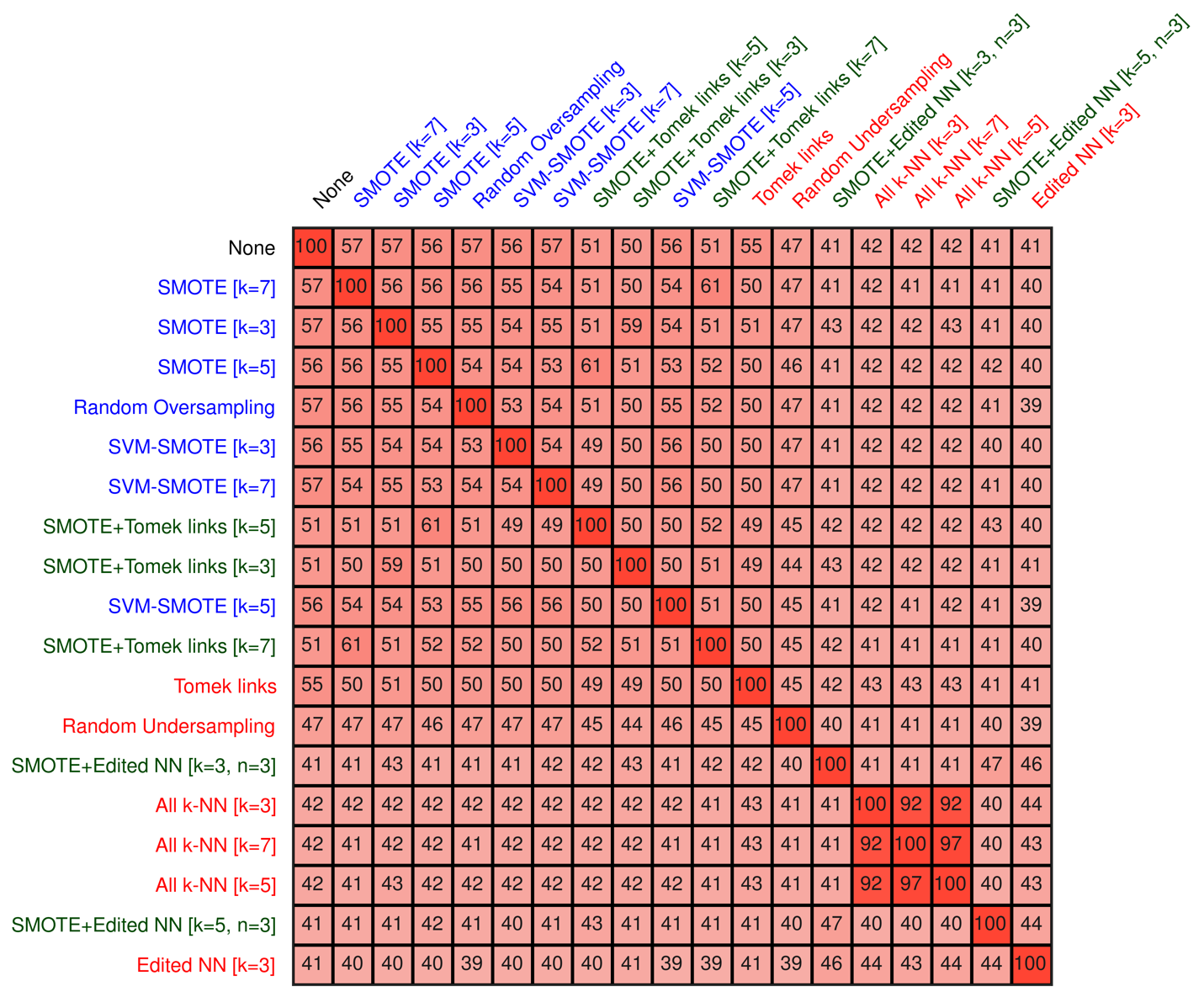


**Figure S2** Features similarity based on Zucknick measure

Similarity among the set of features selected by the resampling methods. The similarity was computed by computing the Zucknick measure and averaging.
